# Exploring factors associated with obesity in Argentinian children using structural equation modeling

**DOI:** 10.1590/0102-311XEN087822

**Published:** 2023-08-11

**Authors:** Ignacio Mendez, María Victoria Fasano, Alicia B. Orden

**Affiliations:** 1 Instituto de Desarrollo e Investigaciones Pediátricas, Buenos Aires, Argentina.; 2 Centro de Matemática La Plata, Facultad de Ciencias Exactas, La Plata, Argentina.; 3 Centro de Salud e Investigaciones Médicas, La Pampa, Argentina.; 4 Consejo Nacional de Investigaciones Científicas y Técnicas, Buenos Aires, Argentina.

**Keywords:** Pediatric Obesity, Healthy Lifestyle, Structural Equation Modeling, Socioeconomic Factors, Obesidad Infantil, Estilo de Vida Saludable, Modelado de Ecuaciones Estruturales, Factores Socioeconómicos, Obesidade Infantil, Estilo de Vida Saudável, Modelagem de Equação Estrutural, Fatores Socioeconômicos

## Abstract

Habits and behaviors related to obesity risk are strongly associated with the family environment and are affected by socioeconomic factors. Structural equation modeling (SEM) allows us to hypothesize on how the relationships between these factors occur and measure their impact. This study aimed to explore the relationship between family socioeconomic indicators and childhood obesity, mediated by habits linked to energy balance, applying a SEM. A cross sectional study was performed on 861 Argentinian schoolchildren aged 6-12 years, from 2015 to 2016. The model included three latent variables: socioeconomic status, healthy habits, and obesity. Socioeconomic status indicators and healthy habits were surveyed by self-administered parental questionnaires, whereas obesity indicators were evaluated with anthropometry. The applied model showed an acceptable fit (NFI = 0.966; CFI = 0.979; RMSEA = 0.048). Socioeconomic status positively influenced parental education, health insurance, and car possession, while negatively influenced crowding (p < 0.001). Healthy habits significantly influenced physical activity, meals frequency, and sleep hours, while negatively influenced sedentary hours and mother’s nutritional status (p < 0.001). Obesity factor positively influenced body mass index, body fat, and waist-to-height ratio (p < 0.001). Finally, socioeconomic status positively influenced health habits, which in turn negatively influenced obesity factor. Healthy habits (especially physical activity and mother’s nutritional status) mediated the relationship between socioeconomic status and child obesity. Further research should include other indicators related to diet, eating habits, and physical activity like neighborhood characteristics.

## Resumen

Los hábitos y comportamientos relacionados con el riesgo de obesidad están fuertemente asociados al entorno familiar y se ven afectados por factores socioeconómicos. El modelo de ecuaciones estructurales (MEE) permite plantear hipótesis sobre cómo se dan las relaciones entre estos factores y medir su impacto. El objetivo del presente estudio fue explorar la relación entre los indicadores socioeconómicos familiares y la obesidad infantil, mediada por hábitos relacionados con el balance energético, aplicando un MEE. Se realizó un estudio transversal con 861 escolares argentinos de 6 a 12 años entre 2015 y 2016. El modelo incluyó tres variables latentes: nivel socioeconómico, hábitos saludables y obesidad. Los indicadores nivel socioeconómico y hábitos saludables se midieron mediante cuestionarios autoadministrados a los padres, mientras que los indicadores de obesidad se obtuvieron mediante antropometría. El modelo aplicado presentó un ajuste aceptable (NFI = 0,966; CFI = 0,979; RMSEA = 0,048). El nivel socioeconómico influyó positivamente en la escolaridad de los padres, en el plan de salud y en el hecho de tener automóvil, mientras que influyó negativamente en el hacinamiento (p < 0,001). La variable hábitos saludables influyó significativamente en la actividad física, en la frecuencia de las comidas y en las horas de sueño, pero influyó negativamente en las horas sedentarias y en el estado nutricional materno (p < 0,001). El factor obesidad influyó positivamente en el índice de masa corporal, en la grasa corporal y en la razón cintura/talla (p < 0,001). Finalmente, el nivel socioeconómico influyó positivamente en los hábitos saludables, que, a su vez, influyeron negativamente en la obesidad. Los hábitos saludables, en especial la actividad física y el estado nutricional materno, moderan la relación entre el nivel socioeconómico y la obesidad infantil. Las nuevas investigaciones deberían incluir otros indicadores relacionados con la dieta, con los hábitos alimentarios y con la actividad física, así como con las características del vecindario.

## Resumo

Hábitos e comportamentos relacionados ao risco de obesidade estão fortemente associados ao ambiente familiar e afetados por fatores socioeconômicos. A modelagem de equações estruturais (MEE) permite levantar hipóteses sobre como ocorrem as relações entre esses fatores e medir seu impacto. O objetivo do presente estudo foi explorar a relação entre os indicadores socioeconômicos familiares e a obesidade infantil, mediada por hábitos ligados ao balanço energético, aplicando uma MEE. Um estudo transversal foi realizado com 861 escolares argentinos de 6 a 12 anos entre 2015 e 2016. O modelo incluiu três variáveis latentes: nível socioeconômico, hábitos saudáveis e obesidade. Os indicadores do nível socioeconômico e de hábitos saudáveis foram medidos por meio de questionários autoaplicáveis aos pais, enquanto os indicadores de obesidade foram obtidos por meio de antropometria. O modelo aplicado apresentou um ajuste aceitável (NFI = 0,966; CFI = 0,979; RMSEA = 0,048). O nível socioeconômico influenciou positivamente a escolaridade dos pais, o plano de saúde e a posse de automóvel, enquanto influenciou negativamente a aglomeração (p < 0,001). A variável hábitso saudáveis influenciou significativamente a atividade física, a frequência das refeições e as horas de sono, mas influenciou negativamente as horas sedentárias e o estado nutricional materno (p < 0,001). O fator obesidade influenciou positivamente o índice de massa corporal, a gordura corporal e a relação cintura/estatura (p < 0,001). Finalmente, o nível socioeconômico influenciou positivamente os hábitso saudáveis, que por sua vez influenciou negativamente a obesidade. Hábitos saudáveis, especialmente atividade física e estado nutricional materno, mediam a relação entre nível socioeconômico e obesidade infantil. Novas pesquisas devem incluir outros indicadores relacionados à dieta, hábitos alimentares e atividade física, como características da vizinhança.

## Introduction

Despite the efforts launched by international health organizations to reduce obesity in children, the rates are still increasing all over the world, exceeding 30% among school-aged children in developing countries ^1^. With few exceptions, the trend has been an increase in obesity rates worldwide. Argentina lacks systematic surveillance programs for children and adolescents that allows to quantify this phenomenon. However, our research team has verified this upward trend with consecutive surveys in the province of La Pampa (Northern Patagonia), since 1990 ^2^. Then, only 15% of schoolchildren were overweight and 2% were obese, which strongly contrasts with the current values of 20% and 12%, respectively ^2^ ^,^ ^3^ ^,^ ^4^. This trend calls for primary and secondary interventions, which require the investigation of the responsible factors.

Several factors have been considered as relevant causes of obesity. At the basic level are the classic “big two”, that is, income and energy expenditure ^5^ ^,^ ^6^. In turn, these components are influenced by multiple variables that make up networks rather than being a uni or bidirectional relationships. Notwithstanding this complexity, the determinants of overweight and obesity are traditionally analyzed using logistic or linear models ^7^ ^,^ ^8^. Although such models are widely used for this issue, they do not facilitate the estimation of causal and indirect effects in a single, integrated equation. Structural equation modeling (SEM), also known as analysis of the structure of covariance and analysis of the latent variable, is a technique that integrates confirmatory factor analysis and multiple regression analysis and considers multiple independent variables and modeling of interactions among variables ^9^.

The shared home environment is probably the closer influence on childhood obesity, which is reflected by strong correlations between parents and children’s habits. Such influence characterizes lifestyles and behaviors transmitted from parents to children with socialization. In fact, the shared environment exposes parents and children to common obesogenic factors, such as unhealthy eating and sedentary habits ^10^. The inﬂuence of socioeconomic status as a determinant or mediator of behavior adds further complexity to our understanding of obesity and its related behaviors ^11^. Eating habits - among others related to obesity - are probably influenced by family income. Although most people know about healthy eating, families with higher socioeconomic status may afford these kinds of foods when compared with lower income families ^12^. Similarly, the influence of the family on energy expenditure and sedentary behaviors of children is related to socioeconomic status, both by access to sports and recreational activities ^13^, as well as several residential environment characteristics that encourage or restrict physical activity ^14^.

The proposal of this study is to introduce a relational model among three latent variables that affect weight gain during childhood: socioeconomic status, healthy habits, and obesity. Our model hypothesizes that higher socioeconomic status is associated with healthier habits and lower childhood obesity. Although this statement is not new, the framework of the indicators may be specific to each community and therefore contribute to adapting local prevention or intervention strategies to reduce obesity.

## Subjects and methods

### Sampling and data collection

The study was conducted in Santa Rosa (La Pampa) between September 2015 and October 2016. We performed a cross-sectional study, based on a two stages cluster sampling. The first stage included neighborhood and school mapping. The second stage consisted of randomly selecting one school per neighborhood. At each school, one division from the first to the sixth grade was randomly selected as well. The sample size was estimated as n = z^2^p(1 - p)/e^2^ with a 95% confidence level (z = 1.96), an overweight and obesity prevalence of 25%, and a 2.5% estimation error. The whole sample comprised 1,366 healthy children aged 6-12 years attending public and private schools.

The research protocol was performed following the guidelines and recommendations of the Ethics Committee of the Pediatric Research and Development Institute, Hospital Sor M. Ludovica, in accordance with national laws and the principles expressed in the *Declaration of Helsinki*. After being approved by the Ministry of Culture and Education of the Province of La Pampa an informed consent was requested from parents or legal guardians, who were previously informed about the objectives and methods of the research. They were also asked to complete a self-administered questionnaire that elicited information on the following domains:

(a) Socioeconomic status: among the variables related to socioeconomic status we consider the maximum educational level of the father and mother (i.e., primary, secondary, or tertiary/university studies); crowding or persons/room ratio; owning a car (categorized by model > 5 years or ≤ 5 years), while no car was scored as 0; and having (1) or not (0) health insurance.

(b) Nutrition and energy expenditure were assessed by daily meal frequency, nocturnal sleep duration, do or not do extra-school physical activity, and daily sedentary hours, i.e., activities without energy expenditure, especially the use of screens.

(c) Parental anthropometry: parents were asked their weight and height to calculate the body mass index (BMI). Then, father and mother’s nutritional status was categorized according to the International Obesity Task Force (IOTF) cut-offs (thinness: < 18.5kg/m^2^; normal weight: 18.5-24.9kg/m^2^; overweight: 25-29.9kg/m^2^; and obesity: ≥ 30kg/m^2^) ^15^. Only a few subjects were classified with thinness, so they were grouped with normal weight.

Anthropometric measurements of children were made at the schools following standardized procedures by a trained observer (A.B.O.). Weight was measured to the nearest 0.1kg with a digital scale Tanita BF-350 (Tanita Corp.; https://www.tanita.com/en/) and height was measured to the nearest 0.1cm with a stadiometer SECA S-213 (SECA; https://www.seca.com/en_us.html), with the head of the child in the Frankfurt plane position. BMI was calculated as weight (kg) divided by squared height (m^2^) and transformed into standard deviation scores (Z_BMI) based on the IOTF reference ^15^. Waist circumference was measured as the smallest circumference between the lower end of the sternum (xiphoid process) and the umbilicus with an inelastic fiberglass tape at the end of normal expiration. Then we estimate the waist-to-height ratio (W/H). Skinfold thicknesses were measured with a Lange caliper at four sites: tricipital (SK_1_), at the mid-point on the posterior line of the upper arm; bicipital (SK_2_) was measured at the same point on the anterior line of the arm; subscapular skinfold (SK_3_) was taken under the inferior angle of the left scapula; and suprailiac skinfold (SK_4_) was measured by lifting a horizontal skinfold above the iliac bone surface. Then, body fat percentage was calculated using the equations of Brook ^16^ based on density (D = 1.1690 - 0.0788*log[Σ^4^_*i=1*_ SK_*i*_ ] for boys, and D = 1.2063 - 0.0999*log[Σ^4^_*i=1*_ SK_*i*_ ] for girls, and according to Siri ^17^ (BF(%) = [(4.95/D) - 4.5]*100).

### Structural equation modeling

SEM allows us to simultaneously evaluate the relationship between indicators (observable or measurable variables) and one or more latent factors (non-observable variables, but indirectly evaluated with indicators), as well as the relationship between latent factors ^18^. These multiple relationships between observable and latent variables are represented by path diagrams, which constitute a hypothesis about the proposed relationships. Thus, SEM is a confirmatory method of that hypothesis. In SEM, a model is said to fit the observed data to the extent that the model-implied covariance matrix is equivalent to the empirical covariance matrix ^19^. Also, a good-fitting measurement model is required before interpreting the causal paths of the structural model.

Our model proposes the existence of relationships between three latent variables: socioeconomic status and healthy habits, both influencing a third called obesity. In the case of socioeconomic status, its indicators are maximum education level of the father, maximum education level of the mother, crowding or persons/room ratio, health insurance, owning a car. Healthy habits is measured by physical activity, nocturnal sleep duration, sedentary hours, meal frequency, mother’s nutritional status, and father’s nutritional status. And obesity is measured by BMI, W/H, and body fat.

### Data analysis

Quantitative variables were examined for normality using the Kolmogorov-Smirnov test: z-score of BMI and W/H had an approximately normal distribution and were reported as mean ± standard deviation (SD), while crowding or persons/room ratio, sedentary hours, and body fat had a log-normal distribution and were reported as geometric mean (GM) with its respective 95% confidence interval (95%CI).

The structural equation modeling was executed using the lavaan 0.6-12 ^20^ and lavaanPlot (https://CRAN.R-project.org/package=lavaanPlot) packages of the R software, version 4.2.1 (http://www.r-project.org). For the parameter estimates, we used weighted least squares method with mean and variance adjusted (WLSMV); the diagonally weighted least squares (DWLS) method was used to estimate model parameters and the full weight matrix to compute robust standard errors, and a mean- and variance-adjusted test statistics. Factor loadings of physical activity, maximum educational level of the father, and BMI were fixed to 1 as an identification restriction. All categorical variables included in the model were binary or ordinal and were declared as such in the model function. When dealing with categorical variables polychoric or polyserial correlations were computed. SEM procedure relies on several statistical tests to determine the adequacy of the model ﬁt to the data. Therefore, once the model was estimated, we verified the compatibility of the model adjustment as well as the obtained estimates. Seven fit indices were reported. The goodness of fit was analyzed with the most commonly used indices: comparative fit index (CFI), Tucker-Lewis index (TLI), relative fit index (RFI), normed fit index (NFI), and incremental fit index (IFI). Indices greater than 0.90 indicate a good fit, and those greater than 0.95 a very good fit ^19^. In addition, two measures related to the unexplained variability of the model were calculated: the root mean square error of approximation (RMSEA) which is an index of the degree to which a conﬁrmatory structure approximates the data being modeled using the variance/covariance matrix, and the standardized root mean squared residual (SRMR). RMSEA values less than 0.06 and SRMR less than 0.08 are considered acceptable. The level of significance was set at p < 0.05.

## Results

The whole sample included 653 boys and 713 girls aged 9.2 ± 1.8 years. After excluding the questionnaires with missing data, the remaining subsample included 861 children (407 boys and 454 girls), aged 9.1 ± 1.8 years. Table 1 shows descriptive statistics regarding the subsample. Table 2 shows the differences with the missing sample. Almost 31% of children were either overweight (20%) or obese (10.6%), as estimated for the total sample ^3^. On average, children had normal W/H values and BMI (z-score > 0) and body fat (> 25%) above the reference values. The education level was variable among the parents, with 25~35.7% of them having a tertiary or university degree. The remaining socioeconomic status data indicated that around 30% of the families had higher levels of socioeconomic status indicators while 25~30% had lower socioeconomic status.

**Table 1:** Variables and descriptive parameters of the study.

Latent variables/Indicator (observables variables)	
Obesity	
BMI (Z-score)	0.72 ± 1.29
W/H	Mean = 0.46 (SD = ± 0.05)
Body fat	Geometric mean: 25.99 (95%CI: 25.44; 26.56)
Socioeconomic status	
Maximum educational level of the father	
Primary	24.0%
Secondary	50.9%
Tertiary/University	25.0%
Maximum educational level of the mother	
Primary	15.9%
Secondary	48.4%
Tertiary/University	35.7%
Crowding (persons/room)	Mean = 1.64 (95%CI: 1.58; 1.69)
Owning a car	
Do not own a car	28.9%
Car ≥ 5 years old	42.4%
Car < 5 years old	28.7%
Health insurance	
Insured	72.9%
Not insured	27.1%
Healthy habits	
Meal frequency (daily)	
2	1.5%
3	14.2%
4	67.8%
5	16.5%
Nocturnal sleep duration (hours)	
≤ 8	21.3%
> 8	78.7%
Physical activity	
Do	63.1%
Do not do	36.9%
Sedentary hours **	
Daily hours looking at screens	Geometric mean = 4.04 (95%CI: 3.93; 4.16)
Father’s nutritional status (kg/m^2^)	
< 24.9	24.9%
25-59.9	50.1%
> 30	25.0%
Mother’s nutritional status (kg/m^2^)	
< 24.9	54.5%
25-59.9	28.4%
> 30	17.1%

95%CI: 95% confidence interval; BMI: body mass index; SD: standard deviation; W/H: waist-to-height ratio.

**Table 2:** Comparison of the known characteristics between the data that is included in the analysis and the excluded or missing data.

Charateristics	Excluded (n = 486)	Included (n = 861)	p-value
Type of school			< 0.001
Public	444 (91.4)	670 (77.8)	
Private	42 (8.6)	191 (22.2)	
Sex			0.570
Male	238 (49.0)	407 (47.3)	
Female	248 (51.0)	454 (52.7)	
Age (years)	8.95 (7.52; 10.77)	9.09 (7.53; 10.60)	0.970
BMI Z-score	0.77 (-0.10; 1.86)	0.57 (-0.21; 1.59)	0.037
W/H	0.46 (0.43; 0.50)	0.45 (0.43; 0.49)	0.002
Body fat percentage	25.83 (20.26; 36.74)	25.46 (20.26; 33.35)	0.130

BMI: body mass index; W/H: waist-to-height ratio.

Note: categorical variables are reported as percentages; quantitative values informed as median (Q1, Q3).

Table 3 shows the results of model fitting based on the SEM. Values of all measures of goodness of fit were within the acceptable range, indicating that the proposed model fitted our data at the 5% significance level. Finally, the measures related to the percentage of variance not explained by the model were RMSEA = 0.048 and SRMR = 0.054. By evaluating all these indicators together, it can be concluded that the overall adjustment of the model was good.

**Table 3:** Goodness of fit indices of the structural equation modelling.

Index	Estimated value	Critical value
RFI (relative fit index)	0.950	> 0.90
IFI (incremental fit index)	0.979	> 0.90
TLI (Tucker-Lewis index)	0.968	> 0.90
CFI (comparative fit index)	0.979	> 0.90
NFI (normed fit index)	0.966	> 0.90
SRMR (standardized root mean square residual)	0.054	< 0.08
RMSEA (root mean square error of approximation)	0.048	< 0.05

**Figure 1:**
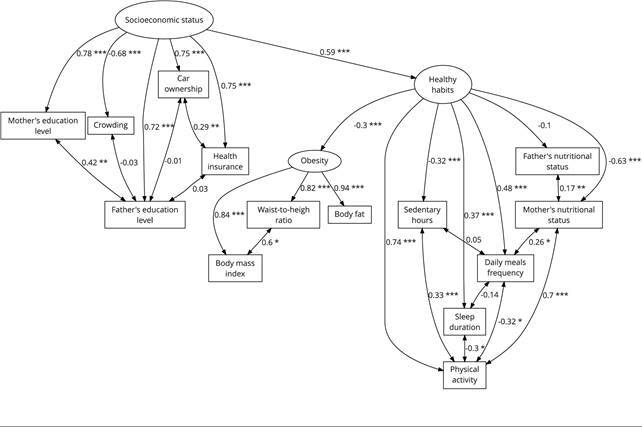
Structural equation modeling (SEM) results of research model.

Note: observed variables are represented by rectangles and latent factors by ellipses. Values in single headed arrows represent standardized estimates. Double headed arrows indicate correlations. * Significant at level 0.05; ** Significant at level 0.01; *** Significant at level 0.001.

Figure 1 and Table 4 show the results of the structural model. Values shown in the figure and table are standardized estimates. It can be observed that socioeconomic status had a positive impact on parental education, health insurance, and owning a car; but negative on crowding or persons/room ratio (p < 0.001). Regarding healthy habits, it was observed to directly and strongly influence physical activity. Likewise, this factor also had a significant positive impact on meal frequency and nocturnal sleep duration, with a negative influence on sedentary hours and mother’s nutritional status (p < 0.001). Regarding the third latent variable, it was observed that obesity had a significant positive impact on the three indicators analyzed (p < 0.001). Finally, socioeconomic status had a significant positive impact on healthy habits, which in turn negatively influenced obesity. In other words, socioeconomic status indirectly impacted obesity through healthy habits.

**Table 4:** Standardized estimates of the model.

Latent variable/Manifest variable	Standardized estimate	95%CI
Healthy habits		
Physical activity	0.736	0.558; 0.913
Nocturnal sleep duration	0.367	0.219; 0.514
Meal frequency	0.479	0.321; 0.638
Mother’s nutritional status	-0.632	-0.766; -0.498
Father’s nutritional status	-0.104	-0.216; 0.009
Sedentary hours	-0.325	-0.435; -0.215
Socioeconomic status		
Maximum educational level of the father	0.725	0.595; 0.854
Maximum educational level of the mother	0.783	0.723; 0.843
Health insurance	0.750	0.666; 0.835
Owning a car	0.747	0.680; 0.813
Crowding or persosn/room ratio	-0.682	-0.743; -0.622
Obesity		
BMI	0.835	0.748; 0.922
W/H	0.822	0.728; 0.916
Body fat	0.937	0.837; 1.037

95%CI: 95% confidence interval; BMI: body mass index; W/H: waist-to-height ratio.

## Discussion

Studies concerning nutrition and health behaviors among children are somewhat scarce in Argentina. Most of the studies on risk factors of obesity use bivariate models. Orden et al. ^3^ combined this method with geometrical techniques such as multiple correspondence analysis. Under this approach, they identified profiles or clusters of individuals with similar BMI status and found that physical activity and calcium intake, as well as sleep, are protective factors of obesity. In contrast, parental obesity, shorter sleep, and lower socioeconomic status have a negative effect on weight gain in childhood. Although the influence of socioeconomic status is undeniable, its pattern of social distribution differs between countries. The global trend is consistent: obesity has increased worldwide as the increase in BMI has shifted from high to low-income sectors ^21^ ^,^ ^22^. Studies in our population have verified such a trend during the last decade, in which obesity is still growing only in low socioeconomic status children ^4^.

The fit model using SEM allowed the evaluation of the influence of socioeconomic status on the health behaviors of children and childhood obesity. Screen time, physical activity, and sleep have been previously identified as relevant factors for children’s obesity risk ^23^ ^,^ ^24^ ^,^ ^25^. We showed that these behaviors are influenced by latent factor “healthy habits”, which acts as a mediator between socioeconomic status and obesity in children. Also, the relationship between higher socioeconomic status and healthy habits could explain better food access, which is associated with better diet quality ^26^ ^,^ ^27^. Contrary to our findings, Géa-Horta et al. ^28^ used SEM to test a model that predicts a positive relationship between child BMI and socioeconomic status. This relationship was mediated by more frequent intakes of obesogenic foods, according to data from a 2006-2007 *Brazilian National Health Survey*. These findings could be explained by rapid changes in the food environment, which, in the past decade, has experienced an increase of ultra-processed, high-energy, and less nutrient-dense foods ^29^. In Argentina, data from the first national nutrition survey collected in 2005 revealed that higher quintiles of income showed a higher proportion of ultra-processed food consumption ^30^. While recent reports from the second national nutrition survey showed that lower-income quintiles had higher consumption of sugar-sweetened beverages and snacks and less consumption of fruits, dairy, and fish ^31^. A food price analysis suggested that, compared to an unhealthy food basket, a healthy one costs around 80% more ^32^.

Many studies examining the relationship between the nutritional status of parents and children have found a strong association between parents and children with obesity. Systematic reviews have observed that both fathers and mothers with obesity have influence on the obesity of their children ^33^ ^,^ ^34^. Similar results were previously found using logistic and multivariate methods ^3^. SEM model allowed us to observe intermediate relationships regarding maternal nutritional status and healthy habits. This may be related to women’s role in childcare and household feeding. Factors that mediate the relationship between the father’s nutritional status and children obesity will need further exploration. 

Previous research has examined the relationship between various behavioral factors associated with eating habits (breakfast, fast food, junk food, and sugary drinks), physical activity, and sleep. Researchers have attempted to identify clusters in which certain behavioral profiles are associated with overweight/obesity. However, there does not appear to be a linear cause-effect process, leading to more complex patterns (for instance children with high levels of physical activity and sedentary behavior), and inconsistencies between studies ^35^. These “mixed patterns” are frequently found in the literature ^36^ ^,^ ^37^ ^,^ ^38^, and they may explain the negative correlation between sleep and physical activity found in our study.

The typification of clusters seems complex, even more so if other variables such as age, sex, and socioeconomic status are considered. It has been proposed that relationships between physical activity, weight status, and screen-based sedentary behaviors change over time and that a critical window exists between 6 and 10 years. During this time, sedentary patterns such as screen time may predict BMI more than physical activity ^39^, highlighting the importance of prospective studies in the evolution of obesogenic behaviors.

According to our results, the impact of healthy habits on obesity is significant and similar to other studies ^11^ ^,^ ^28^. While the impact may seem “small”, this is expected given that childhood obesity is determined by a myriad of biological, behavioral, environmental, and physiological factors that impact at different levels (individual, household, community, society) ^40^. In low and middle-income regions like Latin America, obesity prevention strategies should acknowledge different levels of interventions that address households with social disadvantages ^3^ ^,^ ^4^ ^,^ ^41^. It is important to understand obesity determinants at a population level; but different population groups may have different associations between the model’s factors, which is reﬂected in the resulting strengths of the path coefﬁcients. This information may be useful to health professionals to tailor interventions to specific population groups.

Among the strengths of our work is the fact that childhood obesity was derived as a latent variable that explained several measured variables (W/H, body fat, and age-standardized BMI). Other studies have mainly used child BMI as a proxy for child obesity, and while BMI is a practical tool to classify individuals with high adiposity, it has been pointed out that BMI alone might have low sensitivity ^42^. Similarly, socioeconomic status showed a strong effect on several observed variables, which indicates a high construct validity to represent households living conditions. In addition, the use of SEM supports our model, which can form the basis for generating hypotheses to be tested in future studies. On the other hand, our findings must be interpreted with caution. The major limitation of the study is not considering the dependency structure due to the lack of information from the sampling process (survey parameters), which could change the obtained estimates. Another limitation is the absence of food intake variables (diet diversity, consumption of fruits vegetables, or ultra-processed foods), as well as factors of the physical environment (green spaces) and family (access, support) related to energy expenditure. However, there are few studies in this field that use structural equation modeling, and to our knowledge, this is the first in Argentina. We believe that this new approach appears to be appropriate to deal with the complexity of obesity, and future research should advance this type of analysis to include even more factors in larger population studies.

Another important limitation is the loss of sample data. Indeed, children with missing data showed higher values of BMI, attended mostly public schools, and had parents with lower educational level. The relationships between public school, low parental education, and childhood obesity, previously described in this population ^3^ ^,^ ^4^, suggest that the associations observed using SEM could be stronger if there were no missing data.

In conclusion, healthy habits, especially physical activity, and mother’s nutritional status mediate the relationship between socioeconomic status and childhood obesity. The low correlation between healthy habits and obesity suggests adding other indicators such as diet, eating habits as well as neighborhood characteristics related to leisure time and physical activity.
